# Correlation of Real Time PCR Cycle Threshold Cut-Off with *Bordetella pertussis* Clinical Severity

**DOI:** 10.1371/journal.pone.0133209

**Published:** 2015-07-17

**Authors:** Shelly Bolotin, Shelley L. Deeks, Alex Marchand-Austin, Heather Rilkoff, Vica Dang, Ryan Walton, Ahmed Hashim, David Farrell, Natasha S. Crowcroft

**Affiliations:** 1 Public Health Ontario, Toronto, Ontario, Canada; 2 Dalla Lana School of Public Health, University of Toronto, Toronto, Ontario, Canada; 3 Department of Laboratory Medicine and Pathobiology, University of Toronto, Toronto, Ontario, Canada; Universidad Nacional de La Plata., ARGENTINA

## Abstract

*Bordetella pertussis* testing performed using real-time polymerase chain reaction (RT-PCR) is interpreted based on a cycle threshold (Ct) value. At Public Health Ontario Laboratories (PHOL), a Ct value <36 is reported as positive, and Ct values ≥36 and <40 are reported as indeterminate. PHOL reported indeterminate results to physicians and public health units until May 2012, after which these results were only reported to physicians. We investigated the association between Ct value and disease symptom and severity to examine the significance of indeterminate results clinically, epidemiologically and for public health reporting. *B*. *pertussis* positive and indeterminate RT-PCR results were linked to pertussis cases reported in the provincial Integrated Public Health Information System (iPHIS), using deterministic linkage. Patients with positive RT-PCR results had a lower median age of 10.8 years compared to 12.0 years for patients with indeterminate results (p = 0.24). Hospitalized patients had significantly lower Ct values than non-hospitalized patients (median Ct values of 20.7 vs. 31.6, p<0.001). The proportion of patients reporting the most indicative symptoms of pertussis did not differ between patients with positive vs. indeterminate RT-PCR results. Taking the most indicative symptoms of pertussis as the gold-standard, the positive predictive value of the RT-PCR test was 68.1%. RT-PCR test results should be interpreted in the context of the clinical symptoms, age, vaccination status, prevalence, and other factors. Further information on interpretation of indeterminate RT-PCR results may be needed, and the utility of reporting to public health practitioners should be re-evaluated.

## Introduction

Pertussis is a highly communicable disease caused by *Bordetella pertussis*. Despite the implementation of vaccination programs, pertussis remains endemic in many countries [1–3]. A resurgence of pertussis incidence worldwide occurred from 2010 to 2012 [4–6], underscoring that pertussis is still a disease of great public health concern.

*B*. *pertussis* is a fastidious organism, and the sensitivity of culture, the traditional diagnostic method, is poor [7–9]. However, laboratory methods for the diagnosis of pertussis have been revolutionized since the introduction of rapid and sensitive polymerase chain reaction-based molecular methods in the 1990s [10].

Public Health Ontario Laboratories (PHOL) currently utilizes an IS*481*-based real-time polymerase chain reaction (RT-PCR) assay for the identification of *B*. *pertussis*. IS*481* is a multi-copy insertion sequence [11], resulting in a very sensitive laboratory assay, with cycle threshold (Ct) values >35 representing as little as one colony forming unit (CFU) per reaction [12]. Ct values <36 are reported as positive and Ct values ≥36 and <40 are considered indeterminate by PHOL. The high sensitivity of this test is advantageous, especially for severe cases where a positive diagnosis can guide rapid treatment, or during outbreaks when disease control and public health action must be rapidly implemented. However, the interpretation and significance of an indeterminate result, especially in older children or adults who present atypically, epidemiologically-linked mildly symptomatic cases, or patients who have some immunity, is less clear. These challenges of interpretation are compounded by other factors that can affect Ct values, such as the time between symptom onset and sample collection, use of antimicrobial therapy, or the quality of the sample collected. Some of this information is often not available in laboratory information systems or public health patient databases.

The objective of this study was to link laboratory data with clinical symptom, severity and demographic data from Ontario’s reportable disease database to investigate the association between Ct values and disease symptoms and severity, in order to better characterize the clinical, epidemiological and public health significance of indeterminate test results. In addition we evaluated the appropriateness of the current Ct value cut-off of 36 for positive samples.

## Materials and Methods

### Laboratory testing

Specimens received at PHOL, the provincial reference laboratory, included nasopharyngeal swabs, nasopharyngeal aspirates, bronchial alveolar lavage specimens, as well as cultured specimens. Specimen collection methods and transport duration was variable, due to the various submitters to the PHOL and the large geographical size of Ontario. Specimens received prior to June 2012 were tested for *B*. *pertussis* by RT-PCR as previously described [13]. Subsequently, specimens were tested by duplex RT-PCR to differentiate between *B*. *pertussis* and *B*. *holmesii* as previously described [14].

### Data collection and case definitions

Reporting of pertussis is mandatory from all laboratories and physicians to the medical officer of health within Ontario’s 36 public health units (PHUs) [15]. Prior to May 28, 2012 both positive and indeterminate PCR tests were reported to PHUs and physicians by PHOL. Due to questions about the significance of indeterminate tests results, following this date only positive results were reported to PHUs, but indeterminate results continue to be reported to the submitting physician. All 36 PHUs in Ontario enter reportable disease information into the Integrated Public Health Information System (iPHIS). iPHIS includes clinical, demographic and epidemiological information on each case, as well as laboratory test results if a test was performed, but data completeness varies. Public Health Ontario manages iPHIS and provides advice to PHUs on disease control issues.

Cases are classified into several categories in iPHIS. Confirmed and probable case classifications are reportable in Ontario [16]. Additional case classifications (‘suspect’, ‘person under investigation’, ‘undetermined’) are not reportable, but are used for case management (S1 File) [16].

### Linkage of PHOL and iPHIS data

Positive and indeterminate *B*. *pertussis* PCR test results from September 2011 to September 2012 were linked to all pertussis case records in iPHIS regardless of case classification from July 1, 2011 to November 1, 2012 using stepwise deterministic linkage. Negative PCR test results were excluded. A longer time period for linkage was employed for iPHIS data to account for the timing of iPHIS data entry, which may occur before or after laboratory testing. Information on clinical symptoms, outcome, and hospitalization were obtained from iPHIS.

### Data analysis

Descriptive statistics including frequency distributions, mean and median calculations, and Wilcoxon Rank-Sum test calculations to compare medians for non-parametric data were performed using SAS (version 9.3, SAS Institute Inc., Cary, NC). Fisher’s Exact tests were performed using Stata (version 12.1, StataCorp, College Station, TX). A Holm-Bonferroni correction [17] was applied to account for the multiple tests of significance that were performed on the same dataset. A logistic regression model was built in SAS 9.3 using a forward-building strategy to characterize the relationship between hospitalization and Ct value, using the hospitalization status of cases as the outcome variable and the Ct value as the explanatory variable. The model was adjusted for, and stratified by age (less than one year of age or one year of age and older) to explore whether confounding or effect modification occurred.

The positive predictive value (PPV) of the PCR test was calculated in Excel. For the gold-standard, true cases were defined as patients who reported ‘whooping paroxysmal cough’ and/or ‘cough with apnea/vomiting’ in iPHIS. To explore whether the PPV of the PCR test would improve by raising the Ct cut-off value for a positive test, the PPV was then calculated using 37, 38 and 39 as the positive test cut-off.

### Ethics

This analysis was performed as a program evaluation and no ethics approval was required. All data was received in an anonymized form.

## Results

### Data linkage and case characteristics

**Fig 1:**
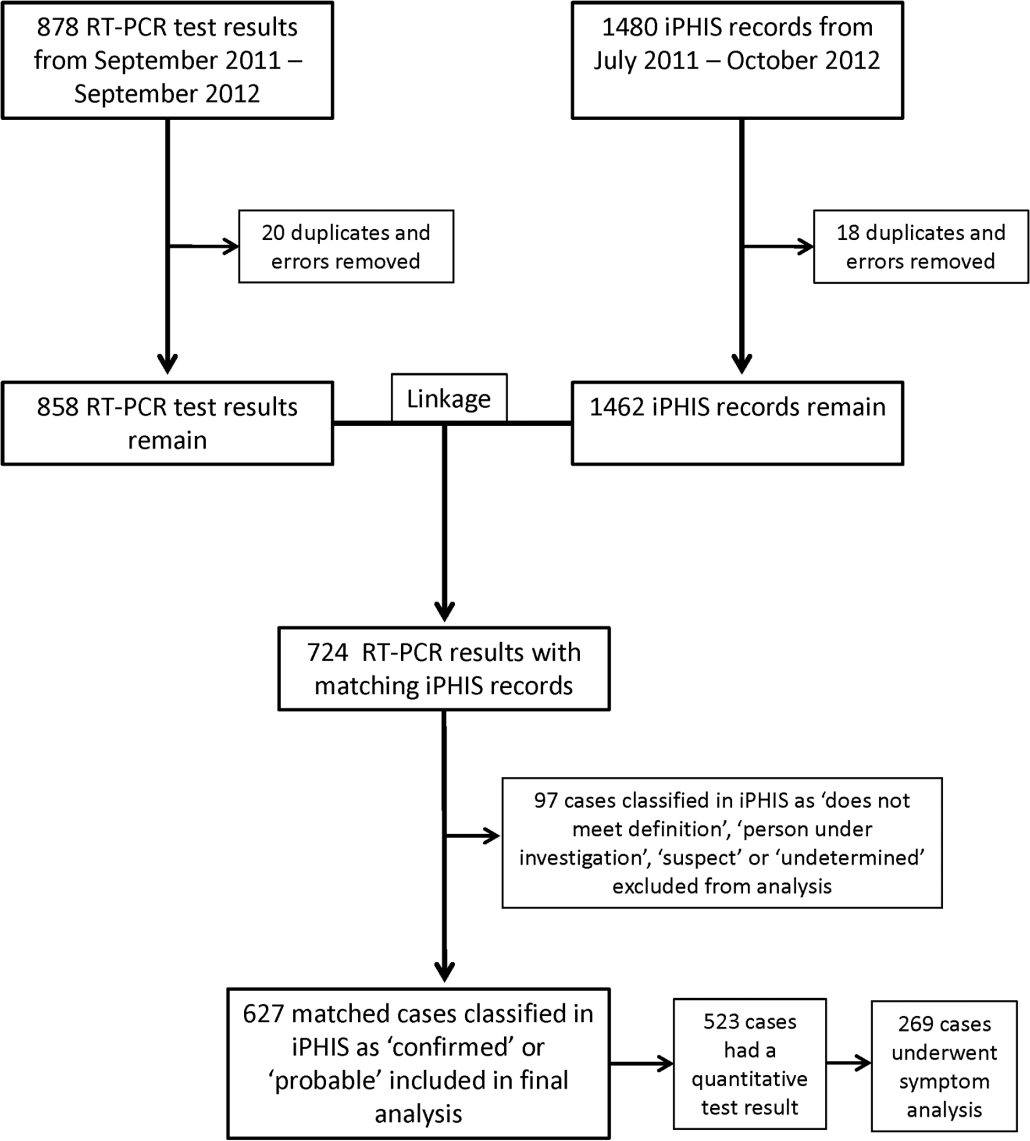
Flow chart showing laboratory test results and iPHIS records used in our analysis. Laboratory results that matched iPHIS cases classified as ‘confirmed’ or ‘probable’ were included in the analysis.

A total of 878 positive and indeterminate *B*. *pertussis* RT-PCR laboratory test results were obtained from PHOL from September 2011 to September 2012. After excluding duplicates and records entered in error, 858 laboratory records remained, five of which were also positive for *Bordetella parapertussis* and one of which was indeterminate for *B parapertussis*. These were compared to 1480 iPHIS pertussis records (1462 after removing duplicates). Following linkage and cleaning, a total of 724 laboratory records (84.4% of all laboratory results) were linked with iPHIS records, leaving 134 laboratory results (15.6%) unlinked (Fig 1). Of the 724 iPHIS-linked laboratory results, 599 were classified in iPHIS as ‘confirmed’ and 28 were classified as ‘probable’, for a total of 627 records. Of these, 557 (88.8%) were RT-PCR positive. This is in contrast to the remaining 97 iPHIS-linked cases, which were classified as ‘does not meet definition’, ‘person under investigation’, ‘suspect’ or ‘undetermined’, of which only 22 cases (22.7%) were RT-PCR positive (Table 1). Since the latter group did not meet the case definition to be a probable or confirmed case, they were excluded from subsequent analysis.

**Table 1:** iPHIS-linked laboratory PCR results by iPHIS case classification (n = 724).

PCR test result	iPHIS case classification						
	Confirmed (%)	Probable (%)	Person under investigation (%)	Does not meet definition (%)	Suspect (%)	Undetermined (%)	Total
**Positive**	554 (92.5)	3 (10.7)	2 (66.7)	20 (22.0)	0 (0)	0 (0)	579 (80.0)
**indeterminate**	45 (7.5)	25 (89.3)	1 (33.3)	71 (78.0)	1 (100)	2 (100)	145 (20)
**Total**	599	28	3	91	1	2	724

Categorical test results were available for all 627 laboratory records, the majority of which (83%) were from nasopharyngeal swabs, and quantitative RT-PCR Ct values were available for 523 (83.4%) records. For positive RT-PCR results (n = 460), the median Ct value was 28.1 (range 13.3–36.0). For indeterminate RT-PCR results (N = 63), the median Ct value was 38.2 (range 36.0–39.5).

**Fig 2:**
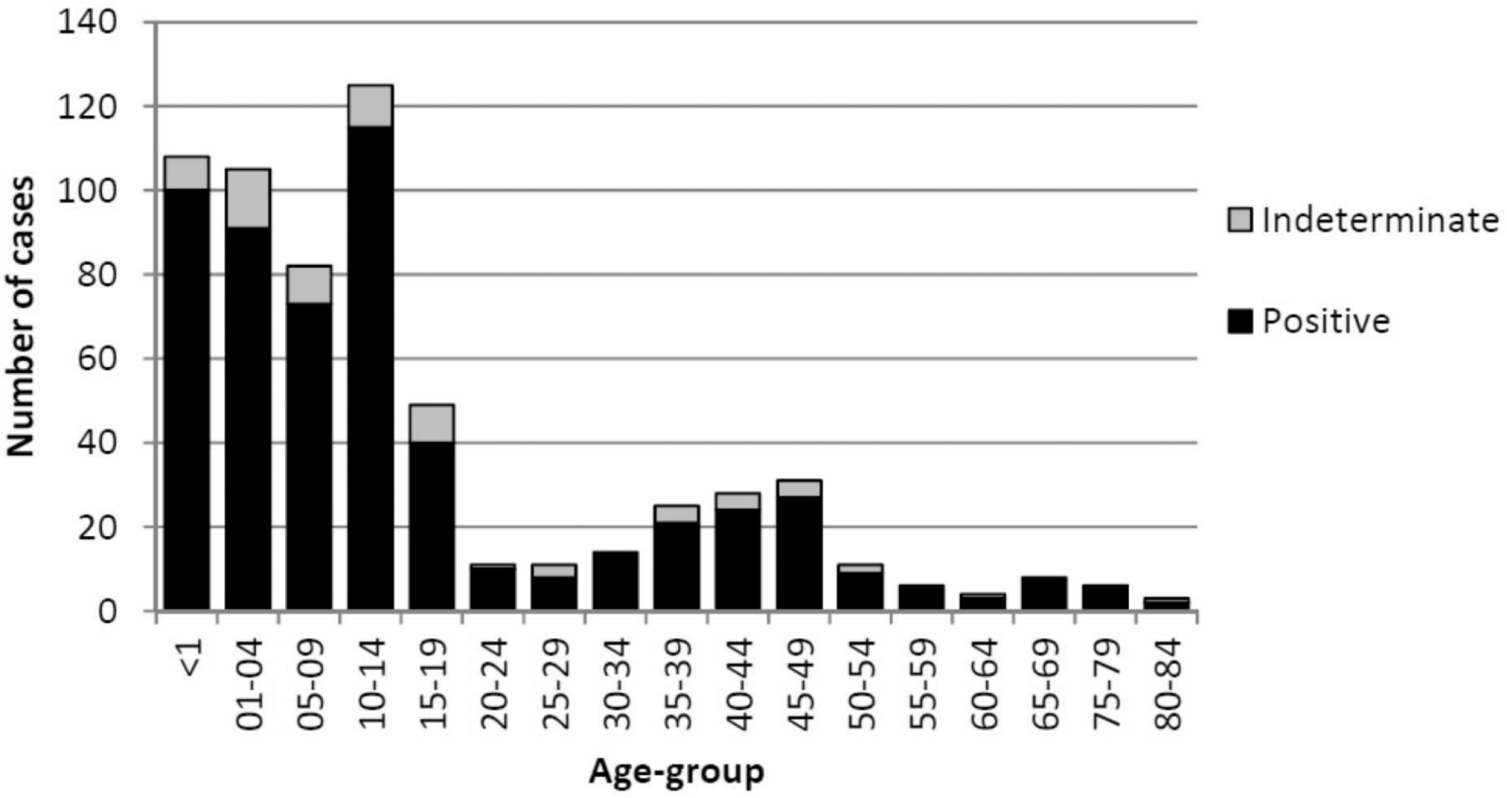
Distribution of PCR Ct values by age-group for iPHIS-linked laboratory records (N = 627). The majority of specimens submitted for laboratory testing from pediatric cases.

The median age of patients with linked laboratory results classified in iPHIS as ‘probable’ or ‘confirmed’ was 10.9 years (range 0–84 years). Patients with RT-PCR positive laboratory results (n = 557) had a median age of 10.8 years (range 0–83.5 years), while patients with RT-PCR indeterminate laboratory results (n = 70) had a median age of 12.0 years (range 0–84 years) (Wilcoxon Rank-Sum p = 0.24). The distribution of laboratory result by age is shown in Fig 2. Patients less than one year of age had a median Ct value of 25.6, while those over the age of one year had a median Ct value of 30.2 (Wilcoxon Rank-Sum p = 0.0003).

### Symptoms and severity of cases

A total of 269 RT-PCR results were linked to ‘probable’ or confirmed’ iPHIS records prior to May 28^th^, 2012. Of these, 255 (94.8%) had at least one clinical symptom recorded in iPHIS (Table 2). The most common symptoms reported were whooping paroxysmal cough (46.1%), coughing with apnea/vomiting (42.4%) and cough (39.0%). After applying a Holm-Bonferroni correction, positive PCR cases were not significantly more likely than indeterminate cases to present with any of the symptoms.

**Table 2:** Distribution of the most frequently reported clinical symptoms among patients with iPHIS-linked laboratory results (n = 269).*

Clinical symptom in iPHIS	Proportion reporting symptoms N (%)	PCR result		Fisher’s exact test p-value**
		Positive (%) (n = 224)	Indeterminate (%) (n = 45)	
**Cough – Whooping paroxysmal**	124 (46.1)	105 (46.9)	19 (42.2)	0.67
**Cough with apnea/vomiting**	114 (42.4)	95 (42.4)	19 (42.2)	1.00
**Cough**	105 (39)	88 (39.3)	17 (37.8)	1.00
**Coryza**	78 (30)	59 (26.3)	19 (42.2)	0.05
**Fever**	50 (18.6)	42(18.8)	8 (17.8)	1.00
**Fatigue**	40 (14.9)	38 (17.0)	2 (4.4)	0.04
**Vomiting**	26 (9.7)	21 (9.4)	5 (11.1)	0.78

*- Analysis is restricted to prior to May 28^th^ 2012. As of May 28^th^ indeterminate cases were no longer reported to public health units and thus data on indeterminate case symptoms is not available after this date.

**- A Holm-Bonferroni correction was applied for interpretation of p-values, after which none of the p-values were statistically significant.

**Fig 3:**
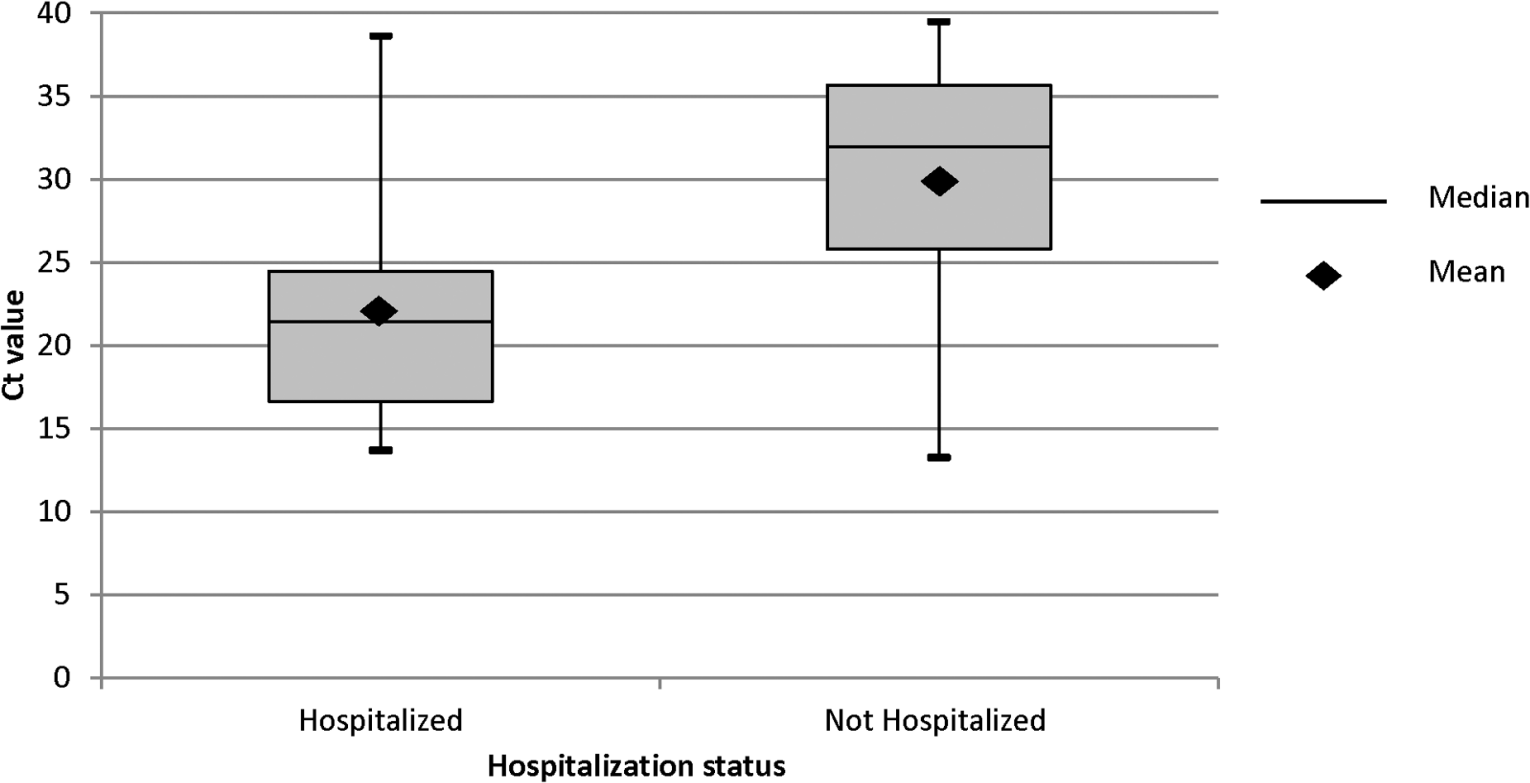
Ct value distribution of iPHIS-linked laboratory samples by hospitalization status of patient. Hospitalized cases had a median RT-PCR Ct value of 20.7 compared to 31.6 for non-hospitalized cases (Wilcoxon Rank-Sum test p < 0.001).

Of 269 patients with RT-PCR results linked to iPHIS records prior to May 28^th^ 2012, 224 (83.3%) had information on both Ct value and hospitalization status, of which 22 (9.8%) were hospitalized. Of these, the majority (18 cases) was under one year of age. Hospitalized cases had significantly lower Ct values than non-hospitalized cases (median Ct value 20.7 vs. 31.6, respectively, Wilcoxon Rank-Sum test p < 0.001) (Fig 3), with the odds of hospitalization decreasing by 12% with every increase of one Ct in the PCR test (odds ratio (OR) 0.88, 95% confidence interval (CI) 0.83–0.94) (Table 3). The age adjusted model showed only a small change in OR after adjusting for confounding (adjusted OR 0.91, 95% CI 0.85–0.97), and the stratified model showed ORs for those under 1 and 1 year of age and older of 0.91 (95% CI 0.84–0.98) and 0.90 (95% CI 0.79–1.04), respectively, suggesting that age did not significantly affect the relationship between Ct value and hospitalization status.

**Table 3:** Odds ratios for the association between RT-PCR Ct value and hospitalization for all cases (N = 224), and stratified by age (N = 54 (<1 year), N = 170 (≥1 year)).

Variable	OR (95% CI)			
	Crude	Adjusted	Stratified by age	
Ct value	0.88 (0.83–0.94)	0.91 (0.85–0.97)	<1 year of age	≥1 year of age
			0.91 (0.84–0.98)	0.90 (0.79–1.04)

### PPV of the RT-PCR test result

To explore the suitability of the current pertussis RT-PCR Ct cut off of 36 cycles, the PPV of the test was calculated to detect a typical clinical case of infection. Since the most indicative symptoms of pertussis are whooping paroxysmal cough and cough with apnea/vomiting, and in iPHIS the case definition for cases classified as ‘probable’ includes both, these symptoms were used as the gold standard. This does not distinguish between primary or secondary infections, although secondary infections are less likely to manifest with typical infections. Using the current RT-PCR Ct cut-off, the PPV of the pertussis RT-PCR was 68.1%. Raising the Ct cut-off to 37, 38 or 39 would change the PPV to 68.3%, 67.8% or 67.1%, respectively; a non-significant change.

## Discussion

Interpretation of high Ct-value RT-PCR test results for *B*. *pertussis* can be challenging due to both the sensitivity of the test and the heterogeneous population that is being tested, ranging from highly symptomatic infants to mildly symptomatic adults with an epidemiological link to a pertussis outbreak. Age, vaccination or immunity status [18], the number of *B*. *pertussis* IS*481* copies [19] and the timing of sample collection [20] are a few of the many factors that affect the test result, making it difficult to conclusively classify patients as infected, and complicating decisions relating to treatment and public health action.

Our analysis found that 88.8% of laboratory-linked confirmed or probable pertussis cases were RT-PCR positive, similar or slightly higher than values reported by others with similar RT-PCR cut-off values [21,22]. This is likely due to the strict case definition that accompanies a ‘confirmed’ or ‘probable’ classification. The median Ct value of cases varied by age, with patients under one year having the lowest Ct values. This is also concordant with other studies [23,24]. In particular, Nakamura *et al*. found that infants had the lowest Ct values, followed by children and then adults, regardless of disease stage. Since Ct value is inversely correlated to bacterial load [25], this finding is not surprising. A higher bacterial load could indicate more severe disease, lower immunity to pertussis, or earlier diagnostic testing [10,18,20,26], all of which are more likely in young children.

The proportions of patients who, according to reportable disease data, reported the symptoms most indicative of pertussis were not significantly different between those with positive RT-PCR tests compared to those with indeterminate results. This was discordant with findings from other studies, in which lower Ct values were associated with the presence of symptoms [10,24]. For example, another study performed in Ontario a few years before ours found a significant correlation between the estimated probability of meeting clinical case definition and Ct value [13,27]. It should be noted, however, that in many studies comparing RT-PCR test result and pertussis symptoms the Ct cut-off was as high as 40 [10,23] or even 50 cycles [24], and the comparison included negative cases. In such a scenario, our Ct value cut-off and range for ‘indeterminate’ cases may be too low and too narrow. Alternatively, these cut-offs and ranges may be appropriate when there is very little pertussis in the population being tested (during inter-epidemic periods or in patients without symptoms) but need to be higher and wider when the prevalence of infection is higher. The lack of correlation between Ct values and reported symptoms may be due to other factors, such as incomplete symptom data entry into iPHIS, misclassification of PCR positive or indeterminate cases that did not meet the ‘probable’ or ‘confirmed’ case definitions into these categories, or true pertussis cases with indeterminate or negative RT-PCR results.

Although PCR positive patients were not more likely to report symptoms as recorded in the iPHIS database, hospitalized patients had a significantly lower Ct value than those who were not hospitalized, indicating that patients with more severe disease may in fact have a higher bacterial load at the time of testing. This finding could also be due to the young age of the majority of hospitalized patients in this study. However, the magnitude of change in the age adjusted logistic regression model as compared to the unadjusted model was small and cannot appreciably explain these effects. It is also possible that the confounding effects of age could not be detected due to the small sample size.

The PPV of the RT-PCR against clinical typical symptoms was not high, and did not change significantly when the Ct cut-off was raised. As seen during the analysis of symptom data, the symptom characteristics of patients with Ct values of <36 (i.e. – positive) were not significantly different than those of patients with Ct values of 36–40 (i.e. – indeterminate), indicating that the cut-off chosen is arbitrary, and indeterminate results could represent true cases.

There are several limitations to our study. Our findings may have been affected by incomplete data entry on cases in iPHIS, especially case symptom data, which was not a mandatory field. We did not validate reported symptoms with medical record review or follow-up with local PHUs. This would particularly impact our results if data quality varied by laboratory test result. We did not analyze immunization data due to incomplete data entry, or data from patients with negative laboratory tests. Linkage of these test results to iPHIS data would not be productive, since patients with negative laboratory tests are unlikely to be classified as ‘confirmed’ or ‘probable’ in iPHIS, and are not normally reported. However, in order to fully describe patients with indeterminate RT-PCR results, their characteristics must be compared to those of patients with both positive and negative RT-PCR results to calculate negative predictive value, sensitivity and specificity. There was also no information available on the duration of symptoms prior to sample collection, and whether empirical antibiotic therapy was commenced before samples were collected for laboratory testing. This may be important as antibiotic treatment would lower the bacterial load in the nasopharynx, resulting in a higher RT-PCR Ct value [28]. Finally, further investigation is warranted into the reasons why some laboratory confirmed cases were missing from the reportable disease database or were not classified as confirmed, although it is recognized that symptoms are required in order to meet either the confirmed or probable case classification.

This data linkage analysis demonstrates the intricacies associated with interpreting the IS*481*-based RT-PCR *B*. *pertussis* test. We found that patients with indeterminate RT-PCR test results were more likely to be older and less likely to have severe illness requiring hospitalization. However, there was no clear division between the epidemiological and clinical characteristics of patients with positive and indeterminate test results. Indeterminate RT-PCR results could therefore represent a true case, but could also be indicative of a single pertussis CFU or residual DNA in the nasopharynx, as would be the case with those who were tested long after symptom onset or previously treated with antibiotics. Test results should therefore be interpreted in the context of a variety of factors, including whether the patient is symptomatic, their age, vaccination status, time elapsed from symptom onset to sample collection, and whether they are epidemiologically linked. The challenge for microbiologists is to express the need for interpretation clearly to clinicians and public health. Further work is required to optimize the RT-PCR test and minimize the proportion of indeterminate tests, which may lead to false negative cases and impact surveillance, vaccine program evaluation and public health control of pertussis.

## Supporting Information

S1 File Ontario case definition for confirmed and probable pertussis cases. (DOCX)
