# Supplementary material for: Correlation of Real Time PCR Cycle Threshold Cut-Off with Bordetella pertussis Clinical Severity
Source: PLoS One. 2015 Jul 17;10(7):e0133209. doi: 10.1371/journal.pone.0133209 (PMC4505870; doi:10.1371/journal.pone.0133209)
Supplement: S1 File — (DOCX) [file pone.0133209.s001.docx]

**Ontario case definition for confirmed and probable pertussis cases**

A confirmed case is defined as a person with laboratory confirmed *B. pertussis* and with clinically compatible signs and symptoms, or a case with clinically compatible signs and symptoms and an epidemiological link to a laboratory-confirmed case (16).

A probable case is defined as a person with cough lasting at least two weeks, in the absence of an appropriate laboratory test and in the absence of an epidemiological link to a laboratory confirmed case. The case must also have one or both of a paroxysmal cough of any duration or a cough with an inspiratory “whoop” (16)
